# Diagnostic prediction models for CT-confirmed and bacterial rhinosinusitis in primary care: individual participant data meta-analysis

**DOI:** 10.3399/BJGP.2021.0585

**Published:** 2022-07-12

**Authors:** Toshihiko Takada, Jeroen Hoogland, Jens G Hansen, Morten Lindbaek, Timo Autio, Olli-Pekka Alho, Mark H Ebell, Johannes B Reitsma, Roderick P Venekamp

**Affiliations:** Julius Center for Health Sciences and Primary Care, University Medical Center Utrecht, Utrecht University, Utrecht, the Netherlands; associate professor, Department of General Medicine, Shirakawa Satellite for Teaching And Research (STAR), Fukushima Medical University, Fukushima, Japan.; Julius Center for Health Sciences and Primary Care, University Medical Center Utrecht, Utrecht University, Utrecht, the Netherlands.; Department of Clinical Epidemiology, Aarhus University Hospital, Clinical Institute, Aarhus University, Aarhus, Denmark.; Department of General Practice, Institute of Health and Society, University Hospital of Oslo, Oslo, Norway.; Head & Neck Surgery, Oulu University Hospital, Oulu, Finland.; PEDEGO Research Unit, University of Oulu, Finland; professor of otorhinolaryngology, Department of Otorhinolaryngology, Head & Neck Surgery, Oulu University Hospital, Oulu, Finland.; Department of Epidemiology and Biostatistics, College of Public Health, University of Georgia, Athens, GA, US.; Julius Center for Health Sciences and Primary Care, University Medical Center Utrecht, Utrecht University, Utrecht, the Netherlands.; Julius Center for Health Sciences and Primary Care, University Medical Center Utrecht, Utrecht University, Utrecht, the Netherlands.

**Keywords:** antibacterial agents, diagnosis, meta-analysis, primary care, sinusitis

## Abstract

**Background:**

Antibiotics are overused in patients with acute rhinosinusitis (ARS) as it is difficult to identify those who benefit from antibiotic treatment.

**Aim:**

To develop prediction models for computed tomography (CT)-confirmed ARS and culture-confirmed acute bacterial rhinosinusitis (ABRS) in adults presenting to primary care with symptoms suggestive of ARS.

**Design and setting:**

This was a systematic review and individual participant data meta-analysis.

**Method:**

CT-confirmed ARS was defined as the presence of fluid level or total opacification in any maxillary sinuses, whereas culture-confirmed ABRS was defined by culture of fluid from antral puncture. Prediction models were derived using logistic regression modelling.

**Results:**

Among 426 patients from three studies, 140 patients (32.9%) had CT-confirmed ARS. A model consisting of seven variables: previous diagnosis of ARS, preceding upper respiratory tract infection, anosmia, double sickening, purulent nasal discharge on examination, need for antibiotics as judged by a physician, and C-reactive protein (CRP) showed an optimism-corrected c-statistic of 0.73 (95% confidence interval [CI] = 0.69 to 0.78) and a calibration slope of 0.99 (95% CI = 0.72 to 1.19). Among 225 patients from two studies, 68 patients (30.2%) had culture-confirmed ABRS. A model consisting of three variables: pain in teeth, purulent nasal discharge, and CRP showed an optimism-corrected c-statistic of 0.70 (95% CI = 0.63 to 0.77) and a calibration slope of 1.00 (95% CI = 0.66 to 1.52). Clinical utility analysis showed that both models could be useful to rule out the target condition.

**Conclusion:**

Simple prediction models for CT-confirmed ARS and culture-confirmed ABRS can be useful to safely reduce antibiotic use in adults with ARS in high-prescribing countries.

## INTRODUCTION

Acute rhinosinusitis (ARS), an inflammation of the nasal cavity and paranasal sinuses lasting <12 weeks,^1^ is a common reason for primary care visits.^2^^,^^3^ Despite evidence that a bacterium can be identified in only a minority of patients with suspected ARS,^4^ antibiotics are frequently prescribed for such patients.^3^^,^^5^^,^^6^ This potentially leads to unnecessary side effects, medical costs, and the emergence of antimicrobial resistance.^7^^,^^8^

To help physicians identify adults with suspected ARS who are most likely to benefit from antibiotics, prediction models for computed tomography (CT)-confirmed ARS defined as the presence of fluid level or total opacification in any sinus, and culture-confirmed acute bacterial rhinosinusitis (ABRS) defined by positive bacterial culture of antral fluid, have been developed.^9^ The rationale for predicting CT-confirmed ARS was that these CT abnormalities are highly indicative for pus or mucopus by antral puncture^10^ and that antibiotics lead to significantly faster and better recovery than placebo in adults with those CT findings.^11^ However, such models have been derived from only one study^9^ that does not provide the opportunity to assess the models’ generalisability, and the sample sizes of the individual studies in this field^10^^,^^12^^–^15 do not meet the required minimum sample size to develop robust models.^16^^,^^17^ In this study, therefore, an individual participant data meta-analysis (IPD-MA) was performed of multiple studies to develop prediction models for diagnosing CT-confirmed ARS and culture-confirmed ABRS in adults presenting to primary care with symptoms of suspected ARS.

## METHOD

The protocol of this IPD-MA has been registered in PROSPERO (CRD42020175659) and has been published elsewhere.^18^ The study was reported according to the PRISMA statement for diagnostic test accuracy studies and the PRISMA-IPD statement.^19^^,^^20^

### Study identification and selection

A systematic search was conducted to identify eligible studies. First, two authors independently reviewed the reference list of a recent systematic review on the diagnostic accuracy of signs and symptoms for ARS.^4^ Next, the PubMed and Embase searches of this review were updated (see Supplementary Table S1) from 1 January 2015 to 1 April 2020. No language restrictions were applied. Two authors independently screened the titles and abstracts of the retrieved records and reviewed the full text of all potentially eligible articles against the following criteria:

- enrolled adults (aged ≥15 years) suspected by their GP of having uncomplicated ARS based on signs and symptoms;
- collected data on readily available signs, symptoms, and/or blood tests; and
- performed CT scan of maxillary sinuses and/or bacterial culture of fluid from antral puncture.^18^

**Table:** How this fits in

Acute rhinosinusitis (ARS) is a very common condition in which it is notoriously challenging to identify patients who could potentially benefit from antibiotic treatment. Existing prediction models for computed tomography (CT)-confirmed ARS and culture-confirmed acute bacterial rhinosinusitis (ABRS) — that is, conditions associated with antibiotic benefit – are based on a single, relatively small dataset that does not provide the opportunity to assess the model performance in other datasets with new patients. In the current individual participant data meta-analyses, prediction models for those two outcomes were developed based on readily available variables (previous diagnosis of ARS, preceding upper respiratory tract infection, anosmia, double sickening, purulent nasal discharge on examination, need for antibiotics as judged by physician, and C-reactive protein [CRP] for CT-confirmed ARS; and pain in teeth, purulent nasal discharge on examination, and CRP for culture-confirmed ABRS). These simple models could be useful to rule out the target condition with fair discrimination and calibration, and hence safely reduce the overall use of antibiotics among adults with symptoms of suspected ARS in high-prescribing countries.

Disagreements about the eligibility of articles were resolved by discussion. This process was complemented by screening references of eligible articles and relevant systematic reviews. In addition, experts in the field were asked if they knew any additional studies. Study authors of eligible articles were invited to provide the de-identified, complete dataset of their original study. The obtained datasets for each of the outcomes of interest were merged.

### Quality assessment of included studies

Two authors independently assessed the methodological quality of the included studies using the Quality Assessment of Diagnostic Accuracy Studies-2 (QUADAS-2) tool.^21^ Disagreements were resolved by discussion.

### Predictors

In the protocol,^18^ the following predictors were considered suitable for inclusion: previous diagnosis of ARS; preceding upper respiratory tract infection (URTI); maxillary pain; pain in teeth; anosmia; cacosmia; double sickening; purulent nasal discharge on examination; overall clinical impression; C-reactive protein (CRP); and erythrocyte sedimentation rate (ESR) (Box 1). To enhance applicability, a decision was taken to discard ESR as it is not frequently used in modern primary care practice. Overall clinical impression could not be used because of unavailability. In the current study the authors also planned to evaluate the added value of duration of illness (>10 days), fever (>38ºC), and severe pain.^18^ However, duration of illness could not be evaluated as it was appropriately recorded in only one study.^10^

**Box 1.** Definition of candidate predictors

**Predictor**	**Definition**
Previous diagnosis of ARS	History of previous ARS episode as reported by patients or based on medical record
Preceding URTI	History of URTI preceding the current episode of suspected ARS as reported by patients or based on medical records
Maxillary pain	Pain (any, unilateral, or bilateral) in maxillary sinus region as reported by patients
Pain in teeth	Pain in teeth as reported by patients
Anosmia	Loss of smell as reported by patients
Cacosmia	Sensation of bad smell as reported by patients
Double sickening	Worsening of symptoms after initial improvement of symptoms (‘two phases in the illness history’) as reported by patients or clinicians
Purulent nasal discharge on examination	The presence of purulent nasal discharge on rhinoscopia anterior or endoscopy
CRP	CRP levels (µl/mL) from blood samples collected by venepuncture (laboratory analysis) or fingerprick (using validated point-of-care testing devices)
Fever (>38°C)	Presence of fever (body temperature above 38°C) as reported by patients or measured by clinician
Severe pain	Pain score >8/10 or equivalent scores using other pain rating scales as reported by patients

*ARS = acute rhinosinusitis. CRP = C-reactive protein. URTI = upper respiratory tract infection.*

### Target conditions

The target conditions of interest were: 1) CT-confirmed ARS defined by a fluid level or total opacification in any maxillary sinus on CT scan; and 2) culture-confirmed ABRS defined by positive growth of bacterial pathogens in fluid from antral puncture.

### Statistical analyses

Details of the statistical analyses are presented in Supplementary Box S1.

#### Handling of missing data

Missing values were imputed using multilevel chained Results of analyses in each of 50 imputed datasets were pooled using Rubin’s rules.^22^

#### Sample size considerations

The maximum number of candidate predictors were calculated based on recent guidance.^17^ For CT-confirmed ARS (*n* = 426, outcome prevalence: 32.9%, *n* = 140), nine predictors could be included in the ordinary logistic regression analysis and 12 in penalised models. For culture-confirmed ABRS (*n* = 225, outcome prevalence: 30.2%, *n* = 68), the maximum number of predictors for the ordinary logistic regression analysis and penalised models were six and eight, respectively.

#### Model development

First, the relationship between CRP and each outcome were assessed (see Supplementary Figure S1) and a decision taken to use log-transformation. Second, heterogeneity in the relationship between individual predictors and each outcome was assessed, by fitting logistic regression models within each study. Next, heterogeneity in model performance across studies was further evaluated by internal–external cross-validation.^23^ Finally, a single logistic regression model for each outcome was fitted on all available data. To reduce model complexity and prevent overfitting, penalised logistic regression modelling was applied.^24^ To assess model performance, optimism-corrected area under the curve (AUC) and calibration slope were evaluated by internal validation using bootstrap resampling.^25^ AUC indicates the ability of a prediction model to differentiate between patients with and without an outcome, ranging between 0.5 (no discrimination) and 1.0 (excellent discrimination). The calibration slope is a measure of agreement between the observed and predicted risk of an outcome. Values <1 indicate that the prediction model is overfitted to the development data.

#### Clinical utility of the derived models

The potential consequences of using the models to select patients for withholding or considering antibiotic treatment based on the estimated risk of the target conditions are shown. In the absence of guidance about the appropriate risk threshold for clinical decision making, information about the consequences of applying various thresholds, that is, ranging from 0.1 to 0.9, are provided.

All analyses were performed using SPSS version 25 (SPSS Inc., Chicago, IL, US) and R version 3.6.3 (R Foundation for Statistical Computing, Vienna, Austria).

## RESULTS

### Study inclusion and study characteristics

**Figure 1.**
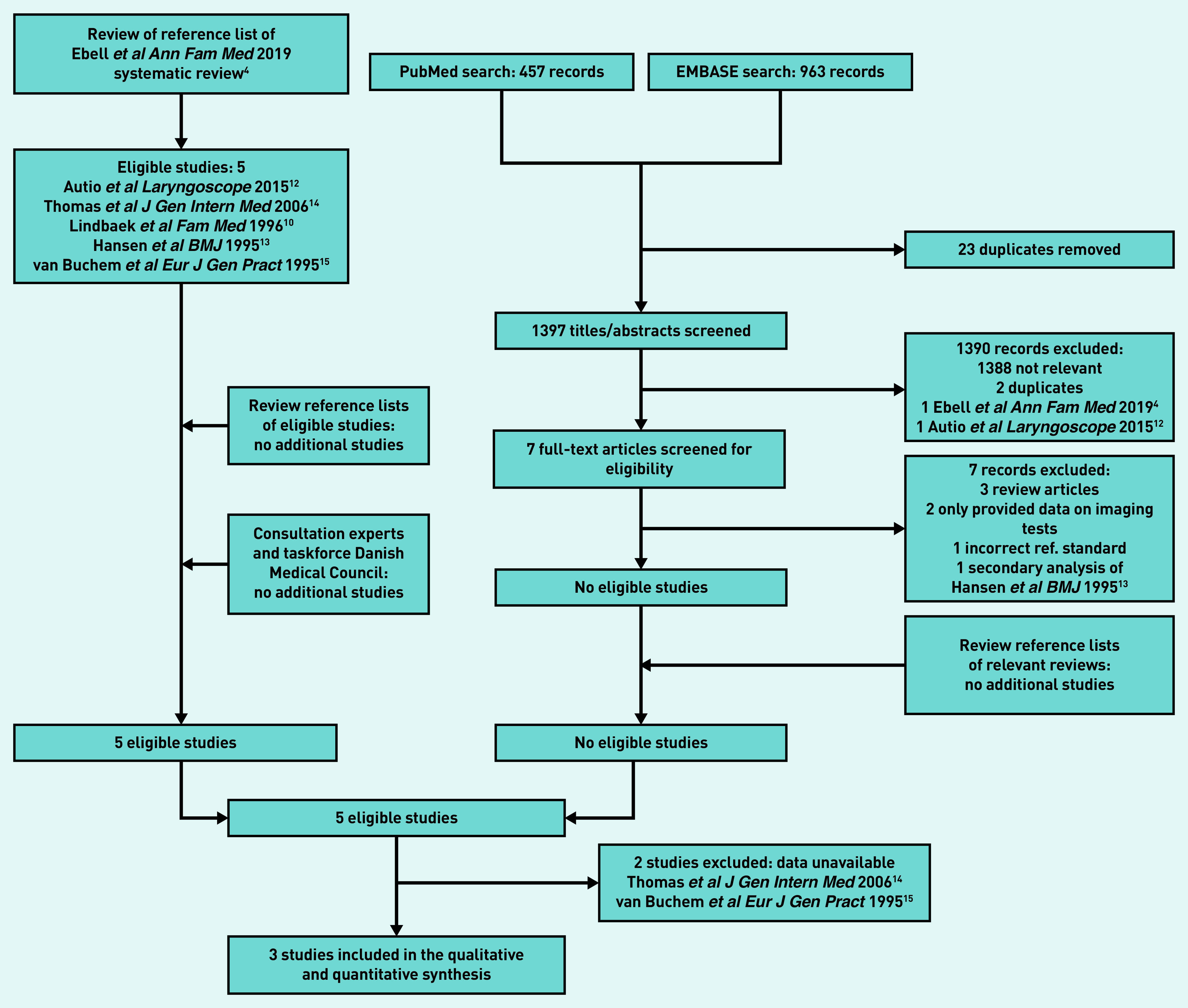
*Study flow.*

Five eligible studies^10^^,^^12^^–^15 were identified from the recent review’s reference list.^4^ No further eligible studies were found from the electronic database searches or additional routes (Figure 1). Two studies^14^^,^^15^ (see Supplementary Table S2) were excluded as the authors were not able to provide IPD, leaving three studies with 426 participants for inclusion.^10^^,^^12^^,^^13^

The characteristics of included studies are shown in Supplementary Table S3. All three studies were conducted in primary care settings, although Autio *et al* included only military recruits.^12^ The other two studies included adults suspected of having ARS; however, Lindbaek *et al* had an additional criterion that antibiotics were considered necessary by the GP.^10^ All three studies were included in the IPD-MA for CT-confirmed ARS^10^^,^^12^^,^^13^ and two with 225 participants for culture-confirmed ABRS.^12^^,^^13^ Patient characteristics are summarised in Table 1. The overall prevalence was 32.9% (*n* = 140, range: 20.6%^13^ to 41.8%^10^) for CT-confirmed ARS and 30.2% (*n* = 68, range: 16.0%^12^ to 34.3%^13^) for culture-confirmed ABRS. The percentage of missing values for each variable is presented in Supplementary Table S4.

**Table 1.** Patient characteristics in each study

	**Autio *et al*^12^ (*n* = 50)**	**Hansen *et al*^13^ (*n* = 175)**	**Lindbaek *et al*^10^ (*n* = 201)**	**Overall (*n* = 426)**
Age, years, median (IQR)	20 (19–20)	35 (27–45)	35 (28–46)	34 (25–44)
Sex, male, *n* (%)	48 (96.0)	52 (29.7)	64 (31.8)	164 (38.5)
Previous diagnosis of ARS, *n* (%)	16 (32.0)	124 (70.9)	119 (59.2)	259 (60.8)
Preceding URTI, *n* (%)	39 (78.0)	148 (84.6)	193 (96.0)	380 (89.2)
Maxillary pain, *n* (%)	37 (74.0)	168 (96.0)	136 (67.7)	341 (80.0)
Pain in teeth, *n* (%)	11 (22.0)	105 (60.0)	98 (48.8)	214 (50.2)
Anosmia, *n* (%)	13 (26.0)	107 (61.1)	143 (71.1)	263 (61.7)
Cacosmia, *n* (%)	15 (30.0)	66 (37.7)	54 (26.9)	135 (31.7)
Double sickening, *n* (%)	45 (90.0)	119 (68.0)	118 (58.7)	282 (66.2)
Pain scale, median (IQR)	0.0 (0.0–3.0)	5.0 (2.0–7.3)	6.2 (3.5–7.8)	5.0 (2.0–7.3)
Purulent nasal discharge on examination, *n* (%)	27 (54.0)	47 (26.9)	84 (41.8)	158 (37.1)
Fever (>38ºC), *n* (%)	0 (0.0)	19 (10.9)	16 (8.0)	35 (8.2)
CRP (µg/mL), median (IQR)	3.1 (1.2–11.6)	18.0 (10.0–38.0)	9.0 (9.0–13.0)	10.0 (9.0–21.0)
CT-confirmed ARS, *n* (%)	20 (40.0)	36 (20.6)	84 (41.8)	140 (32.9)
Culture-confirmed ABRS, *n* (%)	8 (16.0)	60 (34.3)	NA	68 (30.2)^a^

a *The proportion is the prevalence in the data combining the studies by Hansen* et al *and Autio* et al*. ABRS = acute bacterial rhinosinusitis. ARS = acute rhinosinusitis. CRP = C-reactive protein. CT = computed tomography. IQR = interquartile range. NA = not available. URTI = upper respiratory tract infection.*

### Quality assessment of included studies

The quality assessment of included studies is summarised in Supplementary Figure S2. Except for ‘flow and timing’, all items were rated as low risk of bias. In two studies,^10^^,^^12^ the risk of bias for ‘flow and timing’ was rated as unclear as around 15% of participants were excluded from the analyses because of missing information.

### Model development

#### CT-confirmed ARS

When the model was fitted within each study, heterogeneity in the relationship between individual predictors was found and each outcome was not substantial (see Supplementary Figure S3). It was therefore decided to pool the three datasets.

Internal–external cross-validation showed substantial heterogeneity, especially in calibration performance between Hansen *et al*^13^ and Lindbaek *et al*^10^ (see Supplementary Figure S4). The most important difference between these studies was that all patients in Lindbaek *et al*^10^ were judged to need antibiotics, whereas this judgement was not applied in Hansen *et al.*^13^ Therefore, the clinical judgement ‘this patient is likely to need antibiotic treatment’ (‘yes’ versus ‘unknown’) as a predictor was added in the current study.

Among the derived models, the penalised model consisting of seven variables showed the best performance with an optimism-corrected AUC of 0.73 (95% confidence interval [CI] = 0.69 to 0.78) and a calibration slope of 0.99 (95% CI = 0.72 to 1.19) (Table 2). The seven variables were:

- previous diagnosis of ARS;
- preceding URTI;
- anosmia;
- double sickening;
- purulent nasal discharge on examination;
- need for antibiotics as judged by physician; and
- log-transformed CRP.

**Table 2.** Regression coefficients of the CT-confirmed ARS prediction model^a^

	**Ordinary logistic regression**			**Penalised logistic regression coefficient**
	**Coefficient**	**Standard error**	***P*-value**	
Intercept	−4.05	0.77	<0.01	−3.52
Autio^b^	1.37	0.51	0.01	1.08
Lindbaek^b^	1.13	0.31	0.00	0.99
Previous diagnosis of ARS	*–*0.15	0.24	0.52	*–*0.14
Preceding URTI	0.56	0.48	0.24	0.41
Maxillary pain	0.06	0.33	0.85	0.00
Pain in teeth	*–*0.13	0.26	0.62	0.00
Anosmia	0.39	0.27	0.14	0.30
Cacosmia	*–*0.12	0.28	0.68	0.00
Double sickening	0.73	0.35	0.04	0.67
Purulent nasal discharge on examination	1.00	0.23	<0.01	0.97
Log-transformed CRP	0.40	0.14	0.01	0.32

a *The ordinary logistic regression model showed an optimism-corrected AUC of 0.72 (95% CI = 0.68 to 0.77) and a calibration slope of 0.85 (95% CI = 0.67 to 1.10), and the corresponding performance of the penalised regression model was 0.73 (95% CI = 0.69 to 0.78) and 0.99 (95% CI = 0.72 to 1.19), respectively.*

b *The study by Hansen et al was a reference category.^13^ The item ‘Lindbaek’ is defined as positive when physicians judge ‘this patient is likely to need antibiotic treatment’.^10^ The item ‘Autio’ is generally defined as negative as a setting including only military patients is not very likely in clinical practice.^12^ ARS = acute rhinosinusitis. AUC = area under the curve. CRP = C-reactive protein. CT = computed tomography. IQR = interquartile range. URTI = upper respiratory tract infection.*

Fever and severe pain did not have any added value. A web calculator of the penalised model is available online (https://pred-model.shinyapps.io/App_ARS_CT).

#### Culture-confirmed ABRS

Between-study heterogeneity could not be adequately evaluated for the model for culture-confirmed ABRS, as only two studies were available with Autio *et al* having only eight events.^12^ In the absence of clear statistical support or objections, in the current study a decision was taken to pool the two datasets.

The penalised model including three variables showed the best performance with an optimism-corrected AUC of 0.70 (95% CI = 0.63 to 0.77) and a calibration slope of 1.00 (95% CI = 0.66 to 1.52) (Table 3). The three variables were:

- pain in teeth;
- purulent nasal discharge on examination; and
- log-transformed CRP.

**Table 3.** Regression coefficients of the prediction model for culture-confirmed ABRS^a^

	**Ordinary logistic regression**			**Penalised logistic regression coefficient**
	**Coefficient**	**Standard error**	***P*-value**	
Intercept	*–*3.21	1.04	<0.01	*–*2.89
Previous diagnosis of ARS	*–*0.29	0.35	0.41	0.00
Preceding URTI	0.55	0.49	0.27	0.00
Maxillary pain	*–*0.11	0.64	0.86	0.00
Pain in teeth	0.85	0.36	0.02	0.73
Anosmia	*–*0.11	0.34	0.75	0.00
Cacosmia	*–*0.06	0.36	0.86	0.00
Double sickening	0.13	0.75	0.87	0.00
Purulent nasal discharge on examination	0.43	0.35	0.22	0.46
Log-transformed CRP	0.55	0.16	<0.01	0.53

a *The ordinary logistic regression model showed an optimism-corrected AUC of 0.68 (95% CI = 0.62 to 0.75) and a calibration slope of 0.74 (95% CI = 0.47 to 1.06), and the corresponding performance of the penalised regression model was 0.70 (95% CI = 0.63 to 0.77) and 1.00 (95% CI = 0.66 to 1.52), respectively. ABRS = cute bacteriala rhinosinusitis. ARS = acute rhinosinusitis. AUC = area under the curve. CRP = C-reactive protein. URTI = upper respiratory tract infection.*

Fever and severe pain did not have any added value. A web calculator of the penalised model is available online (https://pred-model.shinyapps.io/App_ABRS).

### Clinical utility of the derived models

**Figure 2.**
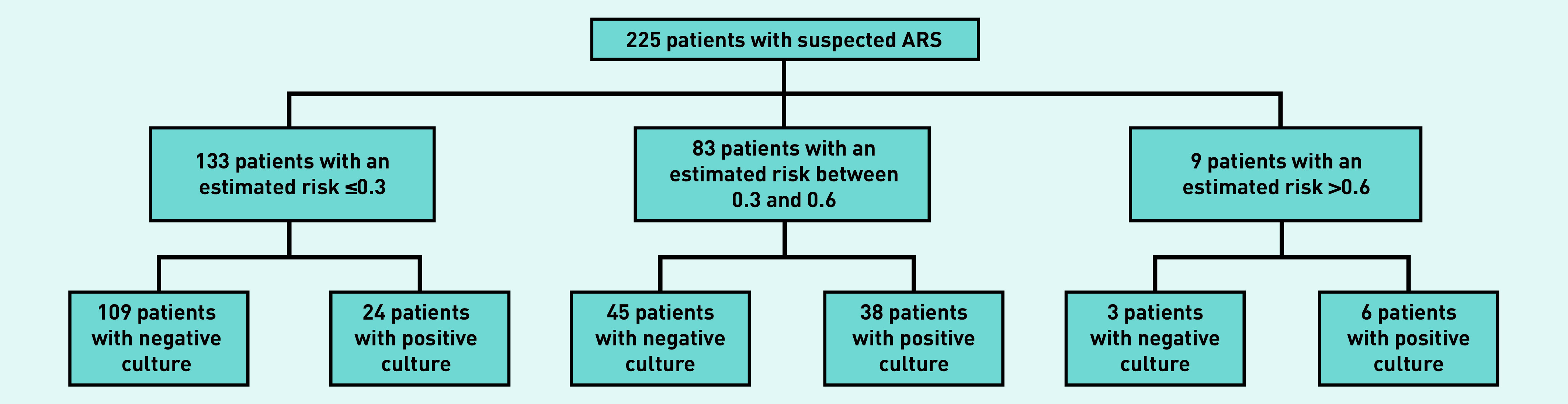
*Example of the consequence of using the derived models in clinical practice. ARS = acute rhinosinusitis.*

The consequence of using the models at various thresholds is illustrated in Supplementary Table S5. Here, for illustrative purposes, the authors have assumed that the culture-confirmed ABRS model is used and antibiotics are withheld in patients with an estimated outcome risk ≤0.3, while considering antibiotic treatment in those with a risk >0.6 (Figure 2). In this scenario, antibiotics would be withheld in 133/225 patients (59.1%, 95% CI = 52.6 to 65.3) at a cost of misclassification — that is, antibiotics are withheld despite having culture-confirmed ABRS — in 24/133 patients (18.0%, 95% CI = 12.4 to 25.4). On the other hand, antibiotics would be considered in only 9/225 patients (4.0%, 95% CI = 2.1 to 7.4), and 3/9 patients (33.3% 95% CI = 12.1 to 64.6) would be misclassified (that is, antibiotics would be considered despite not having culture-confirmed ABRS). This would leave a substantial group of patients (36.9%, *n* = 83/225) having an intermediate risk (between 0.3 and 0.6) and still posing a diagnostic challenge. Also, validation in further datasets is required before adoption of these models in daily practice.

## DISCUSSION

### Summary

In this diagnostic IPD-MA, models were developed with moderate performance for predicting CT-confirmed ARS, defined by a presence of a fluid level or total opacification in any maxillary sinus, and culture-confirmed ABRS, defined by positive growth of bacterial pathogens in fluid from antral puncture. The CT-confirmed ARS model consisted of seven variables (previous diagnosis of ARS, preceding URTI, anosmia, double sickening, purulent nasal discharge on examination, need for antibiotics as judged by physician, and CRP), whereas the model for culture-confirmed ABRS consisted of only three variables (pain in teeth, purulent nasal discharge on examination, and CRP). Clinical utility analyses showed that both models could be particularly useful for ruling out the target condition, and thereby withholding antibiotics in a substantial number of patients at a cost of relatively few misclassified patients.

### Strengths and limitations

To the authors’ knowledge, this is the first IPD-MA, using state-of-the-art methodology, to develop generalisable prediction models for CT-confirmed ARS and culture-confirmed ABRS, target conditions associated with antibiotic benefit in adults presenting to primary care with suspected ARS.

Still, for full appreciation of the derived models, some limitations deserve attention. First, despite the authors’ efforts in the current study to obtain all available data, data from two studies^14^^,^^15^ were unavailable for inclusion. Thus, the number of available studies and participants was relatively small. Particularly for the culture-confirmed ABRS model, between-study heterogeneity could not be adequately evaluated as there were only two available studies.^12^^,^^13^

Second, although focusing on studies conducted in primary care, the prevalence of the target conditions varied substantially across studies likely owing to differences in eligibility criteria. For the CT-confirmed ARS model, in the current study it was necessary to include a predictor ‘this patient is likely to need antibiotic treatment’ (‘yes’ versus ‘unknown’) to reduce heterogeneity. Individual physician’s subjective judgement of this predictor might affect the stability of the model performance.

Third, because of the limited sample size, the number of candidate predictors for developing the model for culture-confirmed ABRS slightly exceeded the sample size guidance, which increased the risk of overfitting. Finally, CT-confirmed ARS and culture-confirmed ABRS was used as a surrogate for antibiotic benefit. However, the presence of these target conditions does not necessarily imply that antibiotic treatment is required. In a previous trial of adults with CT-confirmed ARS, patients allocated to antibiotics were more likely to report symptom improvement after 10 days than those receiving placebo (86% versus 57%, respectively).^11^ Albeit this result indicates that antibiotics have beneficial effects among patients with CT-confirmed ARS, it also means that a large number of patients with positive CT findings may recover spontaneously. Similarly, people with culture-confirmed ABRS can spontaneously recover without antibiotic treatment. Given the indirect association between antibiotic benefit and those two target conditions, the derived models are less suitable for ruling in the target conditions and thereby guiding which patients require antibiotics. Conversely, the models can be useful for ruling out the need for antibiotics as it is very unlikely that antibiotics are beneficial in patients without any signs of fluid level or total opacification on CT scan or those with negative bacterial culture of antral fluid.

### Comparison with existing literature

Previous prediction models were derived from only one study with insufficient sample size.^9^ In addition, predictive information of continuous variables such as CRP was not fully incorporated in previous models.^26^

### Implications for research and practice

Despite recommendations in existing practice guidelines to consider antibiotics only for patients with prolonged or severe symptoms,^27^^,^^28^ antibiotics are commonly prescribed in patients with ARS.^3^^,^^5^^,^^6^ By providing an absolute risk estimate of CT-confirmed ARS and culture-confirmed ABRS the derived models have the potential to guide GPs in high-prescribing countries such as the US and the UK to safely reduce antibiotic prescriptions. Both models have the potential to be implemented in daily practice as they consist of readily available variables. For CT-confirmed ARS these are: 1) previous diagnosis of ARS; 2) preceding URTI; 3) anosmia; 4) double sickening; 5) purulent nasal discharge on examination; 6) need for antibiotics as judged by physician; and 7) CRP. For culture-confirmed ABRS these are: 1) pain in teeth; 2) purulent nasal discharge on examination; and 3) CRP.

For ease of use in clinical practice, the model for culture-confirmed ABRS is simpler than the CT-confirmed ARS model. Furthermore, it does not rely on subjective predictor assessment. However, as the models have been derived from a relatively small IPD set, uncertainty of model estimation and its performance remains. As a result, evaluation of the models’ performance outside the context of this IPD set is still warranted before implementation in everyday practice. In addition, the optimal risk thresholds for ruling out the target condition as a proxy for withholding antibiotic treatment are likely to differ across countries because of variation in medical resource accessibility, clinicians’ prescribing habits, and patient perceptions and demands. Establishing the optimum thresholds in adults with clinically diagnosed ARS, as previously reported for community-acquired pneumonia,^29^ has the potential to assist GPs with clinical decision making in their own setting.

In conclusion, in this IPD-MA, prediction models were developed with fair discrimination and calibration for target conditions associated with antibiotic benefit based on readily available variables. Both models have the potential to assist GPs to rule out the target condition and thereby safely reduce antibiotic prescriptions in high-prescribing countries, but this has to be confirmed in future external validation and impact studies.

## Data

Data will be available to researchers who provide a methodologically sound proposal to achieve the aims in the approved proposal. Proposals should be directed to the corresponding author to gain access to the data. Data requesters will need to sign a data-sharing agreement.
